# 

**Published:** 1890-08

**Authors:** 


					﻿.AAJG-TJST, 1890-
OLD SERIES
VoL xxv
NEW SERiUS
Vol. VIJ^Wo. 6.
& I8S5 O’

Hedical^IurW
JodrnaE



H. V. M. MILLER, M.D., LL.D.
VIRGIL 0. HARDON, M.D.
F. W. McRAE, M.D,, M’n’g-’ng Ed.
'PUBLISHED BY JAMES P.HARRISON&Cgg
tap __________i- Atlanta,ga. Ja
SUBSCRIPTIONS *2. SO A YEAR.
				

